# Therapeutic potential of N-acetylcysteine in acrylamide acute neurotoxicity in adult zebrafish

**DOI:** 10.1038/s41598-019-53154-w

**Published:** 2019-11-11

**Authors:** Melissa Faria, Eva Prats, Cristian Gómez-Canela, Chuan-Yu Hsu, Mark A. Arick, Juliette Bedrossiantz, Manuel Orozco, Natàlia Garcia-Reyero, Tamar Ziv, Shani Ben-Lulu, Arie Admon, Leobardo Manuel Gómez-Oliván, Demetrio Raldúa

**Affiliations:** 1 Institute for Environmental Assessment and Water Research (IDAEA-CSIC), Jordi Girona, 18, 08034 Barcelona, Spain; 2 Research and Development Center (CID-CSIC), Jordi Girona, 18, 08034 Barcelona, Spain; 3 Department of Analytical Chemistry and Applied (Chromatography section), School of Engineering, Institut Químic de Sarrià-Universitat Ramon Llull, Via Agusta 390, 08017 Barcelona, Spain; 4 Institute for Genomics, Biocomputing & Biotechnology (IGBB), Mississippi State University, Starkville, MS USA; 5 Laboratorio de Toxicología Ambiental, Facultad de Química, Universidad Autónoma del Estado de México, Paseo Colón intersección Paseo Tollocan s/n. Col. Residencial Colón, 50120 Toluca, Estado de México Mexico; 6 Environmental Laboratory, US Army Engineer Research and Development Center, Vicksburg, MS USA; 7 The Smoler Proteomics Center and the Department of Biology, Technion-Israel Institute of Technology Haifa, 32000 Haifa, Israel

**Keywords:** Neurotoxicity syndromes, Pharmacodynamics

## Abstract

Two essential key events in acrylamide (ACR) acute neurotoxicity are the formation of adducts with nucleophilic sulfhydryl groups on cysteine residues of selected proteins in the synaptic terminals and the depletion of the glutathione (GSx) stores in neural tissue. The use of N-acetylcysteine (NAC) has been recently proposed as a potential antidote against ACR neurotoxicity, as this chemical is not only a well-known precursor of the reduced form of glutathione (GSH), but also is an scavenger of soft electrophiles such as ACR. In this study, the suitability of 0.3 and 0.75 mM NAC to protect against the neurotoxic effect of 0.75 mM ACR has been tested *in vivo* in adult zebrafish. NAC provided only a mild to negligible protection against the changes induced by ACR in the motor function, behavior, transcriptome and proteome. The permeability of NAC to cross blood-brain barrier (BBB) was assessed, as well as the ACR-scavenging activity and the gamma-glutamyl-cysteine ligase (γ-GCL) and acylase I activities. The results show that ACR not only depletes GSx levels but also inhibits it synthesis from NAC/cysteine, having a dramatic effect over the glutathione system. Moreover, results indicate a very low NAC uptake to the brain, probably by a combination of low BBB permeability and high deacylation of NAC during the intestinal absorption. These results strongly suggest that the use of NAC is not indicated in ACR acute neurotoxicity treatment.

## Introduction

Acrylamide (ACR), a water-soluble type-2 alkene, is used in different processes in the paper, textile, and cosmetics industry, as soil conditioner in mineral processing and mining, and as chemical sewer in grouting operations^1^. ACR induces neurotoxicity by depleting cellular glutathione (GSx) levels and by forming Michael type adducts with functionally critical nucleophilic sulfhydryl thiolate groups on cysteine residues of proteins in the synaptic terminals^2,3^. The loss of function of some relevant proteins directly involved in the synaptic vesicles recycling finally results in an altered synaptic function^3^. ACR poisoning in human is characterized by gait abnormalities, skeletal muscles weakness, numbness of the extremities, and other symptoms related to polyneuropathy^4,5^. Currently, medical treatment of ACR poisoning is still symptomatic, and only the mildly affected patients undergo a full recovery^6^.

N-acetylcysteine (NAC), a precursor of L-cysteine and GSx, is a chemical widely used in humans as a mucolytic agent and for the treatment of paracetamol overdose. As the unsaturated carbonyl structure of ACR is a soft electrophile, the use of nucleophilic scavengers like NAC in the treatment of ACR poisoning has been proposed^3,7^. Once NAC is inside the cells, cytosolic acylase I deacetylates NAC to cysteine^8^, a substrate for gamma-glutamylcysteine ligase (γ-GCL), the rate-limiting enzyme of the GSx biosynthetic pathway^9^. Then, the cysteine formed by deacetylation is available for GSx synthesis by the cells. Thus, the replenishment of GSx levels induced by NAC should counteract the reported depletion of GSH levels in ACR poisoned animals. The protective effects of NAC against ACR systemic toxicity have already been demonstrated *in vivo*, with an increase in GSH levels and a decrease in the malondialdehyde levels in plasma, liver and small intestine of rats^10,11^. However, *in vivo* NAC failed to protect rats from ACR neurotoxicity^12^, and reports on the ability of NAC to cross the brain-blood barrier (BBB) are contradictory (reviewed in Samuni, *et al*.^13^). Thus, the suitability of NAC to protect *in vivo* the brain against ACR poisoning still needs to be clarified.

Zebrafish (*Danio rerio*) is a vertebrate model species increasingly used in neurotoxicology^14,15^. Recently, we have developed an ACR acute neurotoxicity model in adult zebrafish by exposing animals to 0.75 mM ACR in water for 3 days^16^. The characterization of this model showed that, as described in mammals, ACR depleted reduced glutathione (GSH) levels and formed adducts with selected cysteine residues of specific proteins in the brain^16,17^.

In this study, we used the adult zebrafish model of ACR acute neurotoxicity to determine the suitability of NAC to protect the brain against ACR toxicity. First, the potential protective effects of NAC on the ACR neurotoxicity were evaluated at the behavioral and molecular (transcriptome and proteome) levels. Then, the ability of this drug to cross BBB was evaluated by different approaches. First, by the BBB parallel artificial membrane permeability assay (BBB-PAMPA), in order to determine the ability of NAC to cross BBB-like phospholipid membranes by passive diffusion, and second, by direct determination of NAC content in the brain. Finally, we evaluated the ability of the drug to recover the depleted GSx stores and to scavenge ACR in the brain of ACR-exposed fish.

## Material and Methods

### Zebrafish experiments

Adult wild-type zebrafish (standard length: 3.2–3.6 cm) were obtained from Piscicultura Superior (Barcelona, Spain) and housed into the CID-CSIC zebrafish facilities for at least 4 weeks before starting the exposures. During this adaptation time, fish were in 40 L tanks with fish water [reverse-osmosis purified water containing 90 mg/L Instant Ocean® (Aquarium Systems, Sarrebourg, France), 0.58 mM CaSO_4_ · 2H_2_O] at 28 ± 1 °C under a 12L:12D photoperiod. The density of fish was usually 30 zebrafish/tank, and always lower than 1 fish/L. They were fed twice a day with flake food (TetraMin, Tetra, Germany).

Working solutions of 0.3 and 0.75 mM NAC (Sigma-Aldrich, St. Louis, MO) were prepared the day of the experiment from a stock of solution of 50 mM NAC freshly prepared in fish water. The pH of the stock solution was adjusted to 7.2 with 4N NaOH.

To develop the model of ACR acute neurotoxicity, adult zebrafish were exposed for 72 h to 0.75 mM ACR (Sigma-Aldrich, St. Louis, MO) in fish water. To analyze the protective effect of NAC, adult zebrafish (≈50:50 male:female ratio) were pre-treated with two different concentrations of NAC for 24 h and then co-exposed to a mixture of 0.75 mM ACR plus NAC at the same concentration used for the pre-treatment, for an additional 72 h (Supplementary Figure S1). The NAC concentrations tested were 0.3 mM and 0.75 mM and total exposure of fish to NAC would be 96 h. Whereas the former is lower than the ACR concentration, the latter is equimolar to the neurotoxicant concentration. Control fish were maintained in fish water under identical conditions. Experiments were conducted in duplicate or triplicate, in 1,5L tanks with 6 fish in each. Medium was changed every day after feeding. Tanks were kept in an incubation chamber (POL-EKO APARATURA Climatic chamber KK350, Poland) set to 28.5 °C and 12L:12D photoperiod. For brain sample collection, fish were euthanized by inducing hypothermic shock in ice-chilled water (2–4 °C). Brain tissue were dissected and stored at −80 °C for further analyses.

Toxicity test of lethal concentration of ACR (1.5 mM) in the presence of NAC (0.3 or 0.75 mM) was conducted under similar co exposure conditions as mentioned above.

All procedures were approved by the Institutional Animal Care and Use Committees at the CID-CSIC and conducted in accordance with the institutional guidelines under a license from the local government (agreement number 9027).

### Analysis of NAC stability

The stability of NAC in fish water under exposure condition was tested by LC-MS/MS. See Supplementary Methods for more details.

### Novel tank test

All testing was performed in an isolated behavioral room at 27–28 °C. Animals (≈50:50 male:female ratio) were brought to the behavioral room one hour before testing began, to acclimate to the environment, and then, behavioral testing was performed between 10:00 and 13:00 h. All fish used in this study were experimentally naïve and all behavioral testing was performed in a blind manner, with observers unaware of the experimental group. In order to avoid any potential tank effect, the experimental group assigned to each tank was switched between trials.

The NTT, used to assess locomotor activity and anxiety, was performed using an experimental setup previously described^16^ (see Supplementary Methods for more details of this assay) Each trial was video-recorded with a GigE camera mounted in front of the experimental tank. Then, videos were analyzed by Ethovision XT 13.0 (Noldus, Wageningen, the Netherlands). First of all, the front of the tank was divided into two equal virtual zones, top and bottom, and the total distance travelled (cm), distance travelled in the top and in the bottom (cm), time spent in the top (s) and latency to top (s) were determined. The Behavioral Observation Research Interactive Software (BORIS) free software^18^ was used to determine the number and duration (s) of freezing bouts.

### RNA and protein extraction

RNA and protein fractions were simultaneously recovered from the same brain samples to be used in parallel for RNA-seq and proteomics analyses. Briefly, frozen samples were homogenized with Trizol (Invitrogen) in a TissueLyser (Qiagen) with stainless steel beads. After centrifugation, upper aqueous phase was used to isolate RNA and the pellet was used to isolate proteins according to manufacturer’s instructions.

### RNAseq

Twenty-four brain samples (8 biological replicates per treatment; control, 0.75 mM ACR, and 0.3 mM NAC + 0.75 mM ACR treatments) were sent to the Vanderbilt Technologies for Advanced Genomics (Vanderbilt University, Nashville, TN) for library preparation and sequencing on the Illumina Novaseq platform. Briefly, RNASeq libraries were prepared using 200 ng of total RNA and the NEBNext® Ultra™ II RNA Library Prep (NEB, Cat: E7765S) per manufacturer’s instructions, with mRNA enriched via poly-A-selection using oligoDT beads. The RNA was then thermally fragmented and converted to cDNA, adenylated for adaptor ligation and PCR amplified. The libraries were sequenced using the NovaSeq 6000 platform (Illumina, San Diego, CA) with 150 bp paired end reads. The sequence reads of the 24 samples were aligned to the Zebrafish transcriptome GRCz11 (NCBI) using Salmon v 0.12.0^19^ producing an estimated transcript expression. Gene-level expression estimates were produce by summing all transcripts estimates for each gene^20^. Genes with low expression (log transformed counts per million averaged across all samples was less than one) were removed from further analysis^21^. The generalized linear model method from EdgeR v3.20.9^22^ was used to test for differential gene expression. Genes with an FDR adjusted p-value < = 0.05 were considered differentially expressed (DE). Moreover, in order to evaluate the protective effect of NAC at the transcriptomic level, we classified the effect as fully rescued (DE in “ACR vs Control”, not DE in “ACR + NAC vs Control”, and DE in “ACR + NAC vs ACR”), partial recovered (DE in “ACR v Control”, not DE in “ACR + NAC vs Control”, not DE in “ACR + NAC vs ACR”, and expression in “ACR + NAC” closer to Control than ACR) and no recovered (DE in “ACR v Control”, DE in “ACR + NAC vs Control”, not DE in “ACR + NAC vs Control”, and fold change of “ACR + NAC vs Control”and “ACR v Control” are the same direction). Fully rescued genes were defined as those genes. Partial recovery genes were, however, those DEGs whose expression level in the ACR + NAC group was no different from the ACR and the control group. Finally, no recovery genes were defined as those DEGs whose expression level in the ACR + NAC group was significantly different of the control, but no different from the ACR group. GAGE v2.28.2^23^ was used to find significantly perturbed Kyoto Encyclopedia of Genes and Genomes (KEGG) pathways using the gene counts method described in the methods manual.

### Proteomic analysis

After a proteolysis step, peptides were analyzed by LC-MS/MS following standard protocols. See Supplementary Methods for more details.

### BBB-PAMPA

BBB-PAMPA Permeability Assay was performed using a protocol based in Rabal, *et al*.^24^. See Supplementary Methods for more details.

### Analysis of NAC in brain samples

The extraction of NAC from zebrafish brain samples was adapted from previous published methods about the determination of metabolites in adults and larvae of zebrafish^16,25,26^. See Supplementary Methods for more details.

### Total glutathione determination

Total glutathione content (GSx = GSH + 2GSSG) was determined following a protocol adapted from Baker, *et al*.^27^. See Supplementary Methods for more details.

### Acylase I activity

Acylase I activity was determined by measuring the formation of L-methionine by deacetylation of N-acetyl-L-methionine (NAM), using a protocol adapted from Uttamsingh, *et al*.^8^. See Supplementary Methods for more details.

### γ-GCL activity

Gamma-glutamyl-cysteine ligase (γ-GCL) activity was measured with a fluorescence based microplate assay adapted from White, *et al*.^9^, which also allows simultaneously measure GSx levels. See Supplementary Methods for more details.

### Statistical studies

Statistical analysis was performed using SPSS software v25 (IBM, USA). Data are presented as the mean ± SEM unless stated otherwise. Pairwise statistical significance was determined with Student’s t-test or Mann-Whitney test; categorical data was analyzed with Pearson Chi-square test; multivariate analysis was conducted as appropriate either with one-way ANOVA followed by Dunnett’s or Tukey’s test as *post hoc* for data with normal distribution and with Kruskal–Wallis test for non-parametric data. The results were considered significant at p < 0.05, unless otherwise indicated.

## Results

### Stability of NAC in fish water

In order to select the NAC exposure system, stability of NAC in fish water was analyzed by UPLC-MS/MS. NAC concentration measured in freshly prepared working solutions presented values very close to the nominal concentrations. However, when these solutions were incubated under experimental conditions (28 °C and 12 L:12D photoperiod), a rapid decline in NAC concentration became evident (Supplementary Figure S2). Thereby, the time-course analysis showed that 24 and 48 h after incubation, the NAC content in the 0.75 mM NAC solution decreased to 0.57 ± 0.06 mM and 0.42 ± 0.10 mM (Kruskal Wallis test, *P* = 0.051; n = 3), respectively. Similarly, the NAC content in the 0.3 mM NAC solution decreased to 0.21 ± 0.06 mM and 0.06 ± 0.06 mM (Kruskal Wallis test, *P* = 0.077; n = 3), respectively. Therefore, a 24 h semi-static exposure system was selected for the zebrafish exposure experiments.

A point of concern in this study was the co-exposure to ACR and NAC in fish water. If NAC exhibited a significant scavenging activity on ACR in the working solution during the co-exposure, this would result in a decrease in the bioavailability of ACR, an undesirable situation for our purpose of assessing the protective effect of NAC at the target tissue. As the scavenging of ACR by NAC should result in a parallel decrease in the free NAC levels, NAC concentrations at the 0.75 mM ACR + 0.75 mM NAC (ACR + NAC_high_) and 0.75 mM ACR + 0.30 mM NAC (ACR + NAC_low_) solutions were determined when the solutions were added (0 h) and just before the renewal (24 h). Supplementary Table ST2 shows that there are no differences in the NAC content between the NAC and the ACR + NAC working solutions at any of the selected times (Mann-Whitney test, *P* = 1.0; n = 3), a result demonstrating that under our experimental conditions, NAC has not a significant scavenging activity on ACR in the working solutions during the co-exposure period.

### NAC provides a concentration-dependent protection against systemic toxicity induced by ACR

In order to assess the therapeutic potential of NAC to protect against the systemic toxicity of a high ACR concentration, adult zebrafish were pre-treated with NAC (0.3 or 0.75 mM) for 24 h and then co-exposed to NAC and 1.5 mM ACR for 72 h (Supplementary Figure S3). The ACR concentration used corresponds to its 72h-LC_100_^16^. Whereas there was a 100% of mortality in the group of animals exposed to 1.5 mM ACR alone, a significant protective effect was already found with the co-exposure to 0.3 mM NAC, with a 40% of survivors [Pearson Chi-square (1) = 7.5, *P* = 0.006]. When 0.75 mM NAC was used, the percentage of survivors increased to 73.33% of the exposed animals [Pearson Chi-square (1) = 17.368, *P* = 0.000031]. These results demonstrate that NAC provides a significant and concentration-dependent protection against the systemic toxicity induced by ACR.

### Partial rescue of the ACR-induced behavioral effects by NAC co-exposure

**Figure 1:**
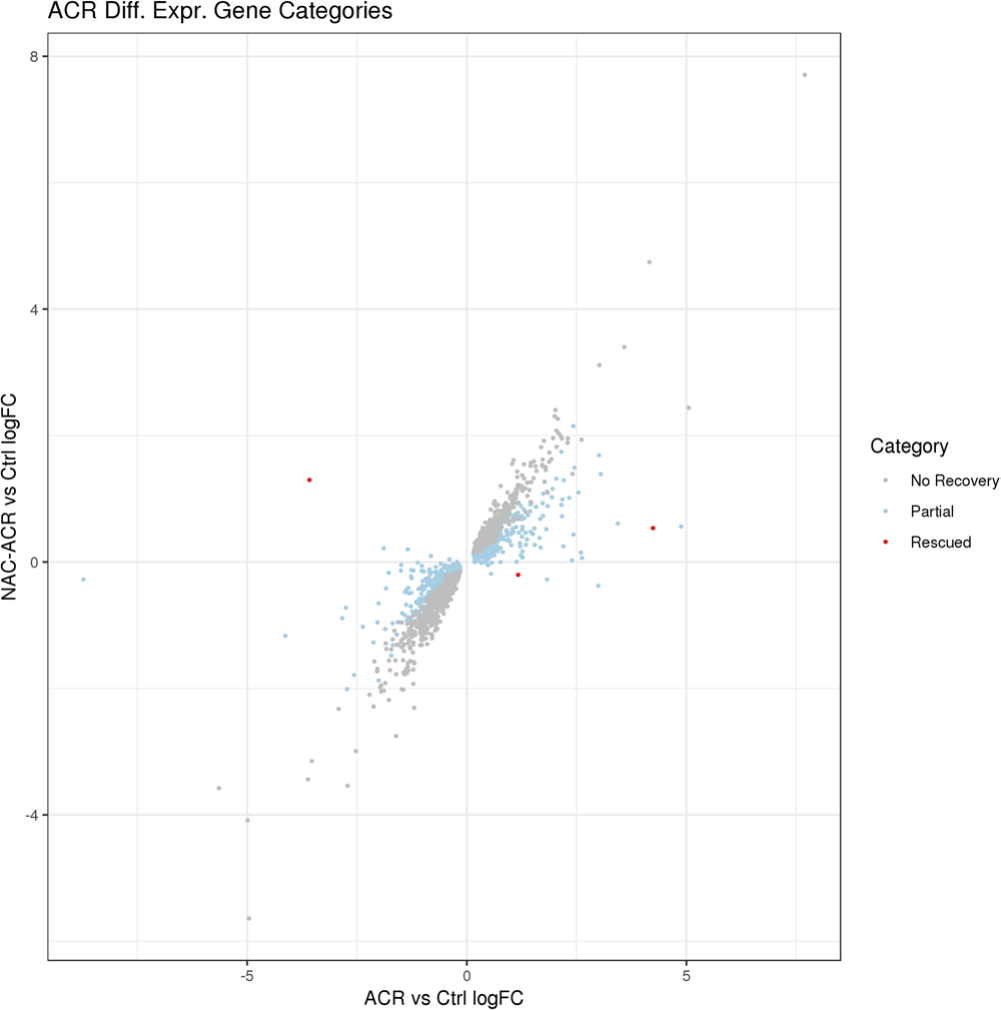
Behavioral parameters of the Novel Tank Test (NTT) for fish from the control, 0.75 mM ACR, 0.75 mM ACR + 0.3 mM NAC and 0.75 mM ACR + 0.75 mM NAC experimental groups. Parameters assessed include: (**A**) total distance travelled, (**B**) distance travelled in the top and in the bottom of the tank, (**C**) time spent in the top, (**D**) latency to enter in the top, (**E**) freezing duration, (**F**) number of freezing bouts, (**G**) time-course of the freezing duration and (**H**) time-course of the freezing bouts. Data reported as mean ± SEM. Different letters indicate significant (*P* < 0.05) differences following one-way ANOVA and Tukey’s multiple-comparison test. Data from 3 independent experiments (n = 18–22).

Results of the Novel Tank Test (NTT) performed with fish from control, ACR, ACR + NAC_low_ and ACR + NAC_high_ experimental groups are shown in Fig. 1 and Supplementary Table ST3. Consistently with a previous report^16^, 72 h exposure to 0.75 mM ACR resulted in hypolocomotion, positive geotaxis and a significant increase in freezing behavior (*P* = 2.51 × 10^−8^, *P* = 2.11 × 10^−8^, *P* = 2.32 × 10^−9^, *P* = 6.6 × 10^−5^ and *P* = 6.56 × 10^−8^ for total distance moved, time spent in the bottom, latency to the top, freezing duration and freezing bouts, respectively; one-way ANOVA with Dunnett’s multiple comparison test; n = 17–22). In this study, the main protective effect of NAC on behavioral changes induced by ACR was in hypolocomotion. Interestingly, although there were no significant differences in locomotor activity between the animals treated with both NAC concentrations, the full rescue of the locomotor activity was only achieved with the lowest NAC concentration, 0.3 mM NAC (Fig. 1A; *P* = 0.0001, *P* = 0.207 when comparing the distance moved by the fish from NAC_low_ grup with those from ACR and control groups, respectively; one-way ANOVA with Tukey’s multiple comparison test; n = 17–23). However, both concentrations of NAC failed to protect against the ACR-induced positive geotaxis, as indicated by the results of the analyses of the distance moved and the time spent in the top of the tank (Fig. 1B,C), as well as the latency to enter in the top (Fig. 1D). Both concentrations of NAC also failed in preventing the time that the animals spent in freezing during the test (Fig. 1E). On the other hand, the number of freezing bouts in ACR-treatment significantly decreased after NAC co-exposure, despite this, the behavioral endpoint was still different from the control fish (Fig. 1H; *P* = 0.010, *P* = 0.011 when comparing the freezing bouts performed by the fish from NAC_low_ grup with those from ACR and control groups, respectively, and *P* = 0.027, *P* = 0.006 when comparing the freezing bouts performed by the fish from NAC_high_ grup with those from ACR and control groups, respectively; one-way ANOVA with Tukey’s multiple comparison test; n = 21–23). Thus, these results indicate that NAC provides only a very limited protection on the behavioral changes induced by ACR.

As the highest protective effect on the altered behavioral endpoints was reached by using a concentration of 0.3 mM NAC, this concentration (NAC_low_) was selected for the further analysis of the potential protective effects of this drug at the molecular level.

### Characterization of the transcriptional changes induced by acute exposure to ACR and NAC in adult zebrafish brain

**Figure 2:**
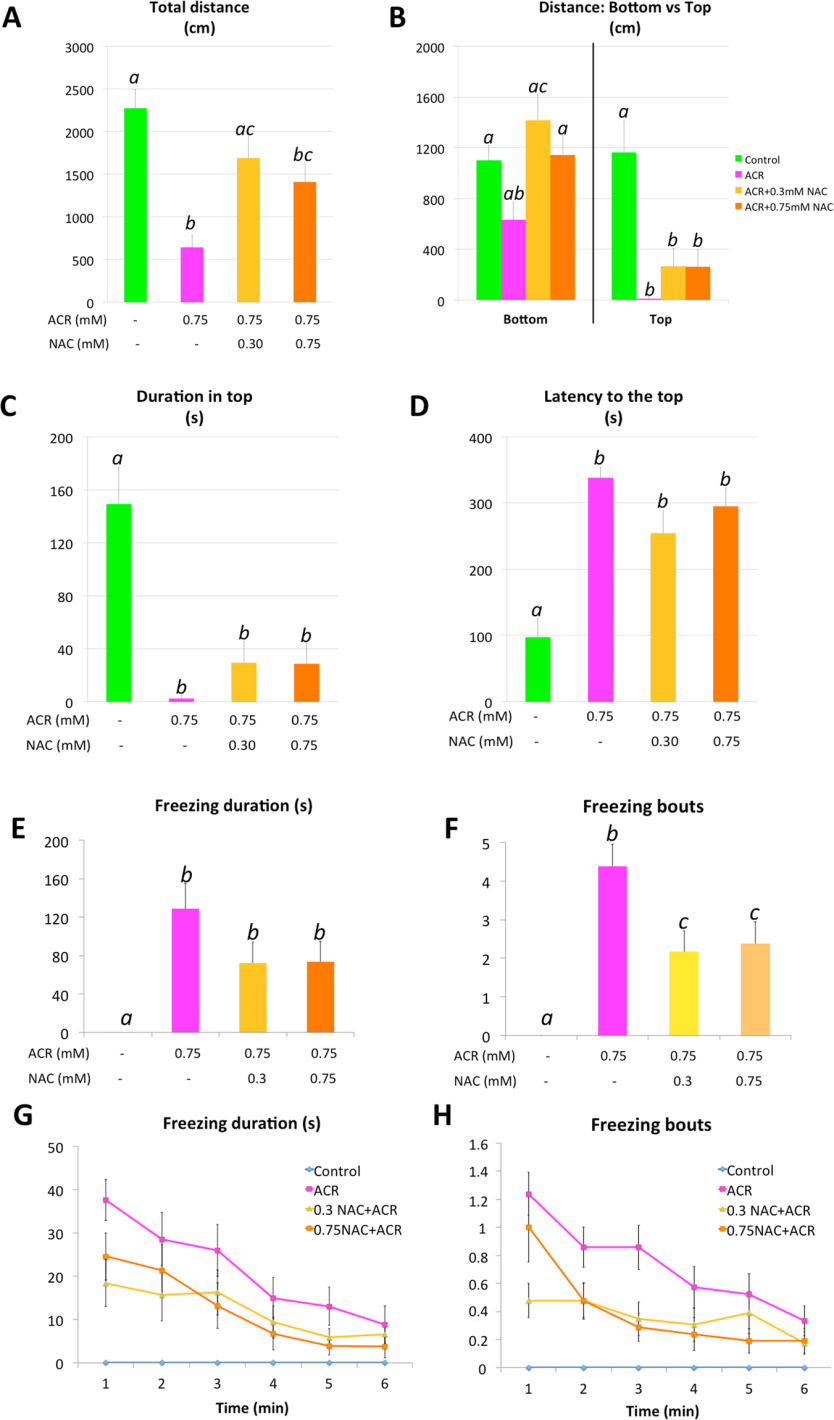
Scatter plot showing the relationship between the fold-change of the expression ACR vs control and NAC-ACR vs control of the differentially expressed genes in the ACR vs control comparison. Genes are colored by their assigned category. Data from 3 independent experiments (n = 8).

A total of 2,359 differentially expressed genes were identified in the brain of ACR-treated zebrafish (Supplementary Dataset S1). Of those, 1,134 were down-regulated and 1,225 were up-regulated. Thirty-five KEGG pathways were significantly enriched after exposure to ACR. Those pathways include steroid biosynthesis, neuroactive ligand-receptor interaction gap junction, or regulation of actin cytoskeleton (Supplementary Dataset S2). In order to understand the therapeutic potential of NAC in the treatment of ACR acute neurotoxicity at molecular level, we compared ACR and ACR + NAC_low_ transcriptomic profiles (Supplementary Dataset S3). Only three genes were fully rescued by NAC, prolyl 3-hydroxylase 3 (*p3h3*), cell surface glycoprotein 1-like, and vicilin-like seed storage protein (Supplementary Dataset S3). This small number of genes detected could also be related to the noise in the system (Fig. 2). Finally, whereas a partial recovery was found in 1,102 DEGs, the expression of 1,254 DEGs was not recovered by NAC (Fig. 2 and Supplementary Dataset S3).

### Effects of NAC on the proteomic changes induced by ACR in the brain

In order to compare the protein profiles of the brain from control, ACR-treated and ACR + NAC_low_ treated fish, eight samples from each group were cleaved and analyzed by LC-MS/MS, identifying 4178 proteins in at least 5 samples in at least one group with at least 2 peptides. When the proteomic profile of control and ACR-treated fish was compared, 80 differential expressed proteins (DEPs; at least 2-fold difference) were identified (Supplementary Dataset S4), and no GO or KEGG annotation enrichment was detected. Then, the potential protective effect of NAC was evaluated, comparing the ACR and ACR + NAC_low_ proteomic profiles. Only 14 of the 80 DEPs were rescued by NAC (Supplementary Dataset S4), although this protective effect might be an artifact representing the noise in the system and not a real effect.

### Limited BBB permeability to NAC

**Figure 3:**
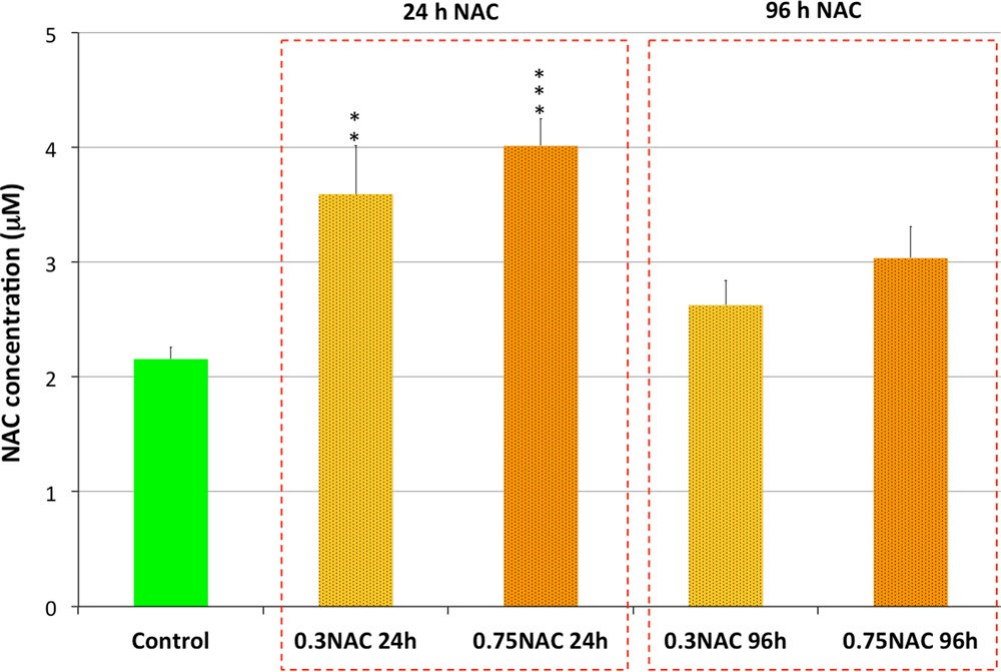
Levels of NAC in the brain of adult zebrafish control and treated with 0.3 mM or 0.75 mM NAC for 24 and 96 h. Although a significant increase in NAC levels was found 24 h after treatment, the levels returned to control values 96 h after treatment. Data reported as mean ± SEM. **P < 0.01, ***P < 0.001; one-way ANOVA with Dunnett’s multiple comparison test. Data from 3 independent experiments (n = 6).

The potential therapeutic value of a drug to protect against the brain toxicity will depend on its ability to cross the BBB. Thus, the unexpected mild protection provided by NAC against the neurotoxic effect of ACR could be due to a limited permeability of NAC across BBB. In order to assess the ability of NAC to cross BBB, two different approaches were used. First of all, the BBB-PAMPA assay was used to determine if NAC is able to cross BBB-like phospholipid membranes by passive diffusion. Whereas propranolol, the positive control^28^, exhibited an expected high permeability (P_e_ > 10 nm/s), the P_e_ for NAC was < 0.1 nm/s, indicating a low permeability of this compound to cross BBB by passive diffusion. Although carrier-mediated active transport of NAC has not yet been reported^13^, NAC levels in the brain of control and NAC-treated zebrafish were determined by UPLC-MS/MS in order to obtain direct evidences of the ability of NAC to cross BBB *in vivo* and in our experimental conditions. NAC is synthesized endogenously, and concentrations of NAC in the range from 23.3 to 137.7 nM have been previously reported in naïve animals^29^. As shown in Fig. 3, whereas the level of endogenous NAC in control fish was 2.15 ± 0.10 μM, the levels of this compound in the brain of animals treated for 24 h with 0.3 or 0.75 mM NAC increased only to 3.59 ± 0.43 μM and 4.01 ± 0.23 μM, respectively. While the observed increase in NAC was statistically significant (*P* = 0.00499 and *P* = 0.00076 for 0.3 mM and 0.75 mM NAC, respectively; one-way ANOVA with Dunnett’s multiple comparison test; n = 6), the NAC content in the brain associated with the exposure represents only the 0.48–0.62% of the nominal concentrations used in the exposure. This very limited increase in NAC concentrations found 24 h after exposure might be related to (1) a low uptake through the BBB or (2) the rapid deacetylation of this drug during the intestinal absorption or directly in the neural tissue. Interestingly, NAC levels returned to the control values after 96 h of continuous exposure (*P* > 0.5 and 0.123 for 0.3 mM and 0.75 mM NAC, respectively; one-way ANOVA with Dunnett’s multiple comparison test; n = 6), suggesting a potential induction of the acylase I activity in the brain in response to the increased NAC levels. In order to test this hypothesis, acylase I activity was determined in the brain and intestine of controls and NAC_low_ - exposed fish. As Supplementary Fig. S4 shows, basal acylase I activity in the intestine (127.15 ± 21.11 pmol/min/mg protein) of control fish was about 6-fold higher than in the brain (21.42 ± 5.39 pmol/min/mg protein), suggesting that most of the NAC absorbed through the intestine was deacetylated *in situ*, reaching the blood circulation as cysteine. Moreover, 24 h and 96 h NAC_low_ - treatments had no significant effect on acylase activity (Supplementary Figure S4). This result indicates that a change in acylase activity was not involved in the decrease of the NAC_low_ levels observed in the brain of the fish 96 h after NAC treatment.

### NAC fails to increase GSx levels in the brain

**Figure 4:**
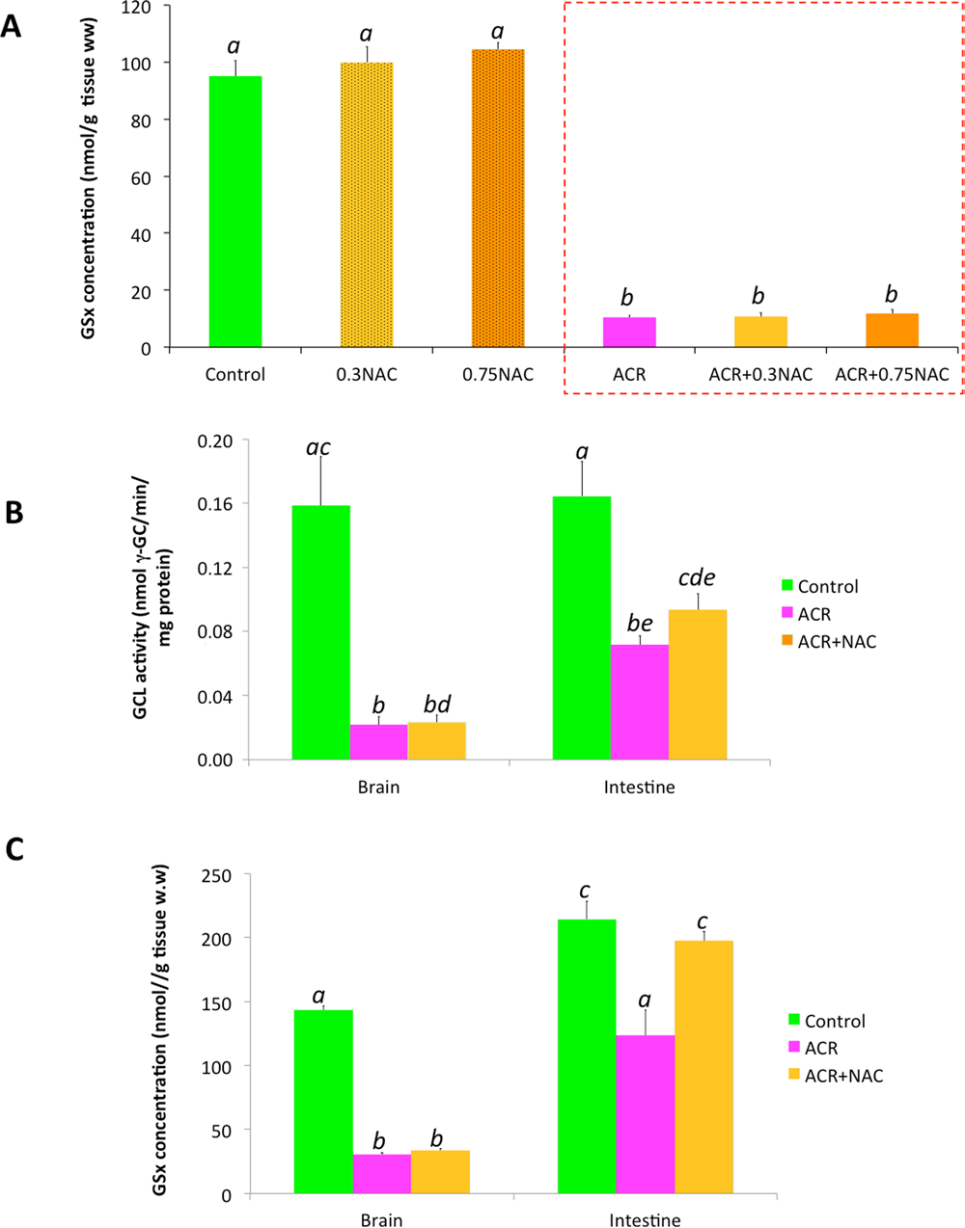
GSx and γ-GCL activity in the adult zebrafish after ACR and ACR + NAC treatments. (**A**) GSx levels in the brain of control, NAC-treated, ACR-treated and ACR + NAC-treated fish. NAC fails to increase GSx levels not only in animals with “normal” GSx levels (control fish), but also in fish with depleted GSx stores (ACR-treated fish). (**B**) Comparison of the γ-GCL activity in the brain and intestine of control, ACR-treated and ACR + NAC_low_-treated fish. (**C**) Comparison of the GSx levels in the brain and the intestine of control, ACR-treated and ACR + NAC_low_-treated fish. Data reported as mean ± SEM. Different letters indicate significant (*P* < 0.05) differences following one-way ANOVA and Tukey’s multiple-comparison test. Data from 3 independent experiments (n = 8–9).

Some reports refer to NAC as a membrane permeable cysteine and GSx precursor^30–32^. Thus, the analysis of the GSx levels in the brain of control and NAC-exposed fish should provide additional information (1) on NAC/cysteine availability at the target tissue, and (2) on the potential rescue of the GSx levels in the treated fish. No significant differences of data variance between groups was observed (one-way ANOVA (F_(2,20)_ = 0.914; *P* = 0.417)). Furthermore, as shown in Fig. 4A, no differences in GSx levels were found in the brain of control (95.10 ± 5.42 nmol GSx/g tissue ww; n = 8), 0.3 mM NAC (99.86 ± 5.56 nmol GSx/g tissue ww; *P* = 0.921, and 0.75 mM NAC (104.50 ± 2.37 nmol GSx/g tissue ww; *P* = 0.430 (Tukey’s multiple comparison test; n = 8) after 96 h exposure. However, the absence of effect of the NAC treatment in the brain of animals with “physiological” levels of GSx might be explained by the presence of feedback mechanisms to maintain stable the cellular GSx levels^33,34^. When GSx levels are depleted, however, an increase in the GSx synthesis is expected^35^. Consequently, we determined the effect of NAC in brains with depleted GSx levels after ACR-treatment. Data variance between groups were found to be significantly different (one-way ANOVA (F_(3,28)_ = 211.807; *P* = 0.000)). The observed difference was due to the dramatic depletion of GSx levels in brain of ACR-treated fish. Figure 4A shows significant decrease of GSx levels in ACR-treated fish compared to the control (10.46 ± 0.79 nmol GSx/g tissue ww and 95.10 ± 5.42 nmol GSx/g tissue ww, respectively (*P* = 6.69 × 10^−13^, Tukey’s multiple comparison test; n = 8). Then, the effect of 0.3 mM or 0.75 mM NAC on GSx levels was determined in the brain of these ACR-treated fish. No differences in the GSx levels were found with 0.3 mM NAC (10.77 ± 1.23 nmol GSx/g tissue ww; *P* = 1.000, or 0.75 mM NAC (11.83 ± 1.35 nmol GSx/g tissue ww; *P* = 0.987, Tukey’s multiple comparison test; n = 8) compared to the ACR-treated fish. Although these results strongly support the hypothesis of a negligible uptake of NAC by the brain, the possibility of a very limited γ-GCL basal activity in the zebrafish brain with respect other organs, was also explored. Therefore, γ-GCL activity was determined in brain and intestine of control, ACR- and ACR + NAC_low_–treated fish. As Fig. 4B shows, brain exhibited a basal γ-GCL activity similar to intestine. Unexpectedly, ACR strongly inhibited γ-GCL activity in both organs. The percentage of inhibition of γ-GCL activity was significantly higher in the brain than in the intestine (86.3 ± 3.2% vs 56.3 ± 3.4% for brain and intestine, respectively; *P* = 0.00002, two-tail Student’s *t*-test; n = 8). Finally, 0.3 mM NAC was able to significantly increase γ-GCL activity inhibited by ACR in the intestine (*P* = 0.04108, one-tail Student’s *t*-test; n = 8), but not in the brain (*P* = 0.4077, one-tail Student’s *t*-test; n = 6–8). When levels of GSx were compared between these two organs, one-way ANOVA analysis revealed significant differences between groups (F(_5,46_) = 56.652; P = 0.000) (Fig. 4C). Levels of GSx in brain were significantly lower than those in intestine *P* = 0.000, Tukey’s multiple comparison test, n = 8). Moreover, GSx-depletion induced by ACR-treatment was more severe in the brain than in the intestine (78.8 ± 0.8% vs 42.4 ± 9.2% decrease respect to the control, for brain and intestine, respectively; *P* = 0.00127, two-tails Student’s *t*-test, n = 9). Finally, Fig. 4C shows that NAC_low_ was able to fully recover the basal levels of GSx in the intestine, but not in the brain.

### NAC doesn’t exhibit ACR scavenging activity in the brain

**Figure 5:**
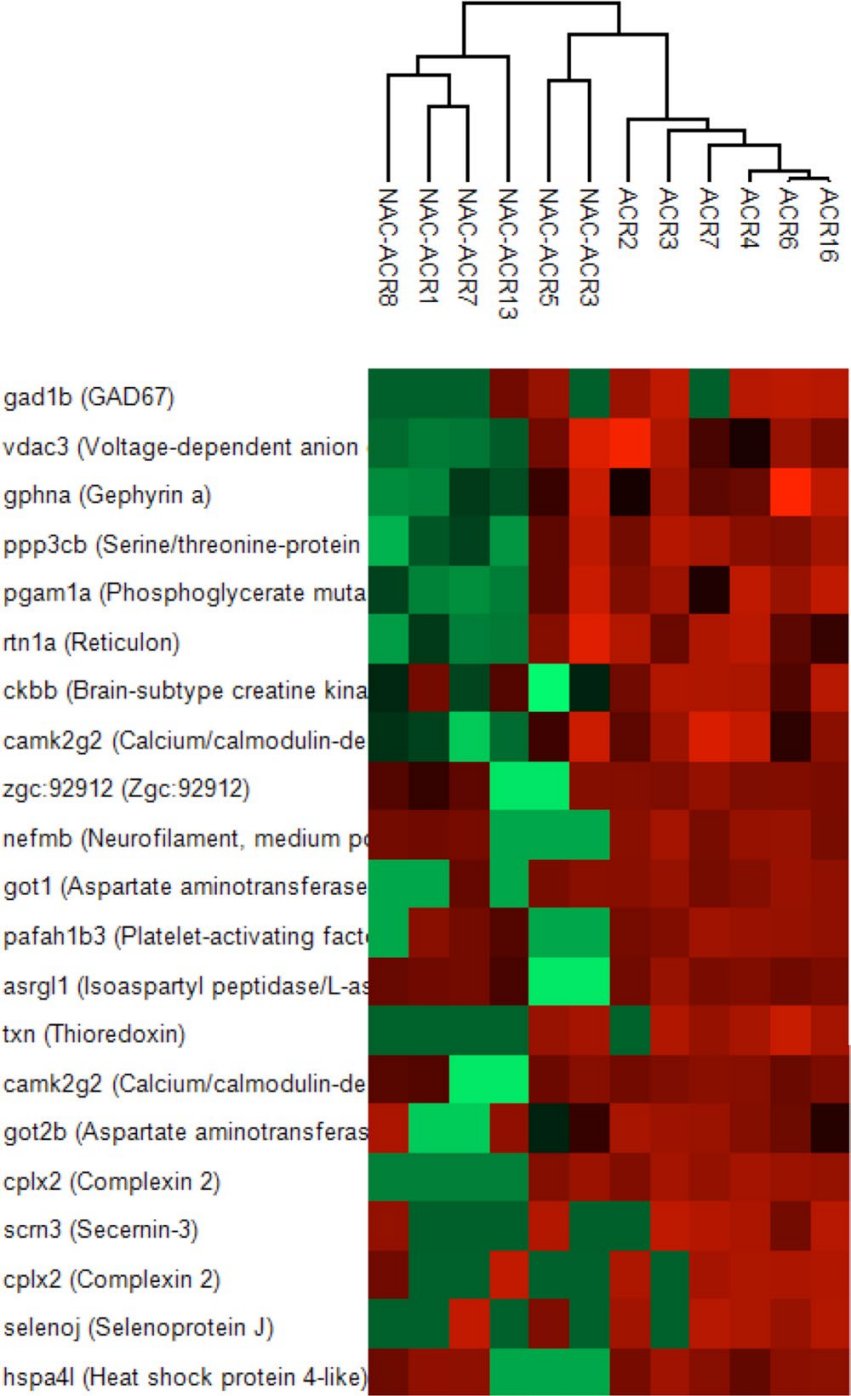
Effect of NAC treatment on the intensities of ACR-modified peptides in the brain of adult zebrafish. Unsupervised clustering was done using the Perseus software and the euclidean correlation and is presented as a heat map of the differential modified peptides. Data from 3 independent experiments (n = 8).

An alternative approach to assess the NAC uptake by the brain is to determine the ACR scavenging activity of this drug in the brain of ACR-treated fish. Our hypothesis was that if this nucleophilic compound were present at significant levels in the brain, it should protect the animals by scavenging ACR and this effect should be reflected not only in a decrease of GSx stores, but also by a significant reduction in the level of ACR-adducts with proteins. Thus, when the presence of covalent acrylamide adducts was analyzed in the brain of ACR-exposed adult zebrafish, modifications on 184 amino acid residues (166 cysteines, 13 lysines and 5 histidines) were identified in different peptides of at least 4 of the 6 treated brains (Supplementary Dataset S5). The peptides with modifications in cysteine residues corresponded with 141 modified proteins, several of them with catalytic activity. Consistent with previous reports many of them are involved in amino acid metabolism, neurotransmission and cell redox mechanisms. Only about 14% of these modified proteins (20 in 141) were significantly protected after NAC_low_ treatment (Supplementary Dataset S5, Fig. 5), a result confirming that NAC concentration in the brain was insufficient to scavenge a significant fraction of the ACR present.

## Discussion

Recently, we developed a new animal model of acute ACR neurotoxicity in adult zebrafish^16^. As described in mammalian models, the zebrafish model of acute ACR neurotoxicity is characterized by the depletion of the GSx stores and the covalent modification of some cysteine residues of selected proteins in the brain^16,17^. NAC, a “membrane permeable” precursor of GSx, is a therapeutic agent used as antioxidant and free-radical scavenging agent, and the use of NAC in a variety of neurological disorders has been proposed^36^.

In this study we have analyzed the potential use of NAC as antidote against the systemic toxicity and neurotoxicity induced by ACR in adult zebrafish. Although ACR is considered to be a selective neurotoxicant at low to moderate concentrations, at high concentrations this compound has been reported to impair the liver^11,37^, kidney^38,39^, and intestinal^11,40^ function. Whereas the 100% mortality found in animals exposed to 1.5 mM ACR for 72 h in this study might be related to a multi organic failure, the increase in survival observed in these animals after pre-treatment and co-exposure with NAC is probably related to the protection of this chemical against the toxic effect of ACR on non neural tissue (Altinoz and Turkoz 2014; Altinoz *et al*. 2015). Interestingly, NAC failed to protect rats from ACR neurotoxic effects^12^, a result consistent with our finding in adult zebrafish, where NAC only provided a mild to negligible protection against ACR neurotoxicity. The low protection provided by NAC to the brain compared to other organs suggests a permeability problem of this compound to cross BBB. Whereas NAC was initially considered as a cysteine and GSx membrane permeable precursor^31,36^, further studies found that at pH 7.4 this compound loses a proton from the carboxyl group, acquiring a negative charge that hinders its transport across BBB by passive diffusion (reviewed at Samuni, *et al*.^13^. Our BBB-PAMPA results have demonstrated a low permeability of NAC to cross BBB by passive diffusion. Moreover, as the active transport of NAC through the membranes has not been described so far, the uptake of NAC by the brain in the exposed fish should be negligible, a prediction consistent with the very low levels of NAC detected in the brain of NAC-treated fish in this study. Whereas the reports on the ability of NAC to cross BBB are contradictory, many of them support a very low permeability of this drug^41–43^. Interestingly, although ACR administration has been reported to increase BBB permeability^44^, in this study co-treatment of fish with NAC and ACR didn’t increase the NAC content in the brain.

In addition to a low permeability of NAC to cross BBB, alternative explanations to the low levels of NAC found in the brain of the treated fish include (1) that most of the NAC is rapidly hydrolyzed to cysteine during the uptake in the intestine and gills, and (2) that NAC crosses BBB but is rapidly hydrolyzed to cysteine into the neural tissue. Cysteine can use different transporters, such as the excitatory amino acid transporter 3 (EAAT3) and the alanine, serine, cysteine transporter (ASCT), to enter into the cells^45^, and the uptake of cysteine by the brain has been estimated to be >500 fold faster than NAC^46^. Thus, cysteine formed during intestinal or gill uptake of NAC could cross BBB, replenishing GSx stores in the brain. Our results on acylase I activity in intestine strongly support this hypothesis. Also the hydrolysis of NAC inside the neural tissue should result in the replenishment of GSx stores. Finally, an alternative explanation to the low levels of NAC found in the brain of ACR + NAC-treated fish is that after crossing BBB, the ACR scavenging activity of the NAC results in a significant reduction in the levels of free NAC. If this hypothesis was true, animals co-exposed with NAC should present a significant decrease in ACR adducts in proteins. However, only a mild decrease in the ACR-modified proteins was found in the brain of the ACR + NAC-treated fish, a result strongly suggesting that only a negligible amount of NAC was able to cross BBB.

Different studies have indicated the ability of NAC, directly or indirectly, to increase GSx levels in brain. Thus, a significant increase in GSx levels was found in the brain of gerbils after intraperitoneal injection of NAC^47^. NAC was also able to recover the GSx levels in the brain of Sprague-Dawley rats treated with 2-cyclohexen-1-one (CHX), a chemical inducing GSx depletion^48^. NAC also recovered the GSx levels in the brain of mice intracerebroventricularly injected with the aggregated amyloid-β-peptide, a treatment inducing GSx depletion^49^. However, our results show that NAC treatment fail to increase GSx levels in the brain of both naïve animals and ACR-treated GSx-depleted animals. In agreement with our results, different reports show the inability of NAC to increase GSx levels in control rats^31,50^ and fish^5^, probably because of the feedback inhibition of the γ-GCL by GSH itself^51^. However, although the inability of NAC to increase GSx stores in the brain of control fish could be explained by the negative feedback of the endogenous GSx, its inability to replenish the depleted GSx stores in the ACR-treated fish is more difficult to understand.

In this study we report for the first time the inhibitory effect of ACR on γ-GCL activity. The inhibitory effect of ACR was significantly stronger in the brain than in the intestine, and NAC-treatment was able to partially rescue this activity only in the intestine. Whereas the transcriptomic analysis shows an up-regulation of the catalytic subunit of glutamyl-cysteine ligase (*gclc*) in response to the GSx depletion, proteomic analysis indicates no changes in protein expression and covalent modification of this protein by ACR adducts. Further studies will be necessary to characterize γ-GCL activity inhibition by ACR and the consequences for the NAC therapeutic action.

The most likely scenario in the brain of the ACR-treated fish is a concomitant depletion of the GSx and the impairment of the GSx *de novo* synthesis by its inhibitory effect on γ-GCL activity. Moreover, most of the NAC absorbed by the intestine is likely deacetylated to cysteine before reaching the blood circulation, and only a small part of the NAC absorbed is able to cross BBB. As γ-GCL is strongly inhibited, uptake of the cysteine generated from NAC doesn’t result in an increase in the GSx synthesis in the brain. Finally, the negligible amount of NAC able to cross BBB is not enough to scavenge a significant portion of ACR accumulated in the brain, and as a result is unable synaptic proteins against ACR adducts formation.

The results presented in this manuscript not only suggest that the use of NAC is not indicated in ACR acute neurotoxicity treatment, but also that the protection of NAC against the other neurotoxic soft electrophiles, as methyl mercury, should be re-evaluated.

## Supplementary information

Supplementary Information

Supplementary Dataset S1

Supplementary Dataset S2

Supplementary Dataset S3

Supplementary Dataset S4

Supplementary Dataset S5

## Data Availability

The mass spectrometry proteomics data have been deposited to the ProteomeXchange Consortium via the PRIDE partner repository with the dataset identifier PXD014228. The sequencing data are archived in the NCBI Short Reads Archive with the BioProject accession number PRJNA545942. The authors declare that all other data supporting the findings of this study are available within the manuscript and its Supplementary Information Files.
