# Publisher Correction: Therapeutic potential of N-acetylcysteine in acrylamide acute neurotoxicity in adult zebrafish

**DOI:** 10.1038/s41598-020-58946-z

**Published:** 2020-02-05

**Authors:** Melissa Faria, Eva Prats, Cristian Gómez-Canela, Chuan-Yu Hsu, Mark A. Arick, Juliette Bedrossiantz, Manuel Orozco, Natàlia Garcia-Reyero, Tamar Ziv, Shani Ben-Lulu, Arie Admon, Leobardo Manuel Gómez-Oliván, Demetrio Raldúa

**Affiliations:** 1 Institute for Environmental Assessment and Water Research (IDAEA-CSIC), Jordi Girona, 18, 08034 Barcelona, Spain; 2 Research and Development Center (CID-CSIC), Jordi Girona, 18, 08034 Barcelona, Spain; 3 Department of Analytical Chemistry and Applied (Chromatography section), School of Engineering, Institut Químic de Sarrià-Universitat Ramon Llull, Via Agusta 390, 08017 Barcelona, Spain; 4 Institute for Genomics, Biocomputing & Biotechnology (IGBB), Mississippi State University, Starkville, MS USA; 5 Laboratorio de Toxicología Ambiental, Facultad de Química, Universidad Autónoma del Estado de México, Paseo Colón intersección Paseo Tollocan s/n. Col. Residencial Colón, 50120 Toluca, Estado de México Mexico; 6 Environmental Laboratory, US Army Engineer Research and Development Center, Vicksburg, MS USA; 7 The Smoler Proteomics Center and the Department of Biology, Technion-Israel Institute of Technology Haifa, 32000 Haifa, Israel

Correction to: *Scientific Reports* 10.1038/s41598-019-53154-w, published online 11 November 2019

Supplementary files containing 5 Supplementary datasets and an additional Supplementary Information File were omitted from the original version of this Article.

This has been corrected in the HTML and PDF versions of the Article.

