# Supplementary material for: Therapeutic potential of N-acetylcysteine in acrylamide acute neurotoxicity in adult zebrafish
Source: Sci Rep. 2019 Nov 11;9:16467. doi: 10.1038/s41598-019-53154-w (PMC6848153; doi:10.1038/s41598-019-53154-w)
Supplement: Supplementary file 1 — Supplementary Information [file 41598_2019_53154_MOESM1_ESM.pdf]

Supplementary Information

Therapeutic potential of N-acetylcysteine in acrylamide acute neurotoxicity in adult zebrafish

Melissa Faria, Eva Prats, Cristian Gómez-Canela, Chuan-Yu Hsu, Mark A. Arick II, Juliette Bedrossiantz, Manuel Orozco, Natàlia Garcia-Reyero, Tamar Ziv, Shani Ben-Lulu, Arie Admon, Leobardo Manuel Gómez-Oliván, Demetrio Raldúa*

*Address correspondence to: Demetrio Raldúa, Institute of Environmental Assessment and Water Research (IDAEA-CSIC), Jordi Girona 18, 08034 Barcelona, Spain. Telephone: +34-93-4006138. E-mail: drpqam@cid.csic.es

Title of file for HTML: Supplementary Information

Description: Supplementary Methods, Supplementary Tables, Supplementary Figures, Supplementary Discussion and Supplementary References

Title of file for HTML: Supplementary Dataset S1

Description: **Differentially expressed genes (DEGs) in the brain of ACR-treated zebrafish compared to the control group**.

Title of file for HTML: Supplementary Dataset S2

Description: **Perturbed Kyoto Encyclopedia of Genes and Genomes (KEGG) pathways in the brain of ACR- and NAC+ACR compared to the control group, as well as the analysis of the protective effect of NAC on the perturbed KEGGs by comparing NAC+ACR vs ACR-treated groups.**

Title of file for HTML: Supplementary Dataset S3

Description: **Evaluation of the protective effect of NAC at transcriptional level**. **The list of the fully rescued, partially recovered and no recovered genes in ACR-treated fish after NAC co-exposure is included.**

Title of file for HTML: Supplementary Dataset S4

Description: **Effect of NAC on the differentially expressed proteins (DEPs) identified in the brain of ACR treated zebrafish. Those proteins significantly recovered by NAC are in red.**

Title of file for HTML: Supplementary Dataset S5

Description: Analysis of the protective **effect of NAC on the formation of ACR-protein adducts in the brain of zebrafish. Proteins significantly protected by NAC are in yellow.**

Supplementary Methods

Analysis of NAC stability

NAC was spiked in zebrafish water at 0.3 and 0.75 mM and were kept at 28 ± 1°C under a 12L:12D photoperiod. Aliquots were analyzed at time 0, 24 and 48 h. Analyses were carried out by direct sample injection using an AcQuity ultra-performance liquid chromatography (UPLC) system equipped with a quaternary pump and connected to a triple quadrupole MS/MS system (Waters, USA). A Synergy Polar-RP column (250 mm x 4.6 mm, particle size 4 µm, Phenomenex, Torrace, USA) was used. The mobile phase consisted in 0.1% formic acid in HPLC water (solvent A) and 0.1% formic acid in MeOH (solvent B). The initial mobile phase composition was 95% A and 5% B, increasing to 30% B in 2 min and to 60% B two minutes later. Then, the initial conditions were regained in 5 min with an equilibration time of 1 min. The flow rate was set at 0.6 mL min^{-1}. To optimize ionization and to establish mass spectral features, individual NAC was first analyzed by flow injection analysis (FIA) in UPLC-MS in positive electrospray ionization (ESI) mode. Full-scan data acquisition was performed scanning from *m/z* 50 to 400. A profile mode was used to determine fragmentation patterns and sensitivity, using a scan time of 2 s with a step size of 0.1 u and a pause between each scan of 2 ms. Optimized parameters were source temperature (from 125 to 150°C), cone voltage (from 5 to 50 V) and collision energy (from 5 to 50 eV). Data were acquired and processed using MassLynx 4.1 software package.

Proteomic analysis

Proteolysis

The protein pellet was washed with 70% acetone and resuspended in 8 M urea, 100 mM ammonium bicarbonate, and 10 mM DTT. Prior to digestion, protein amount was estimated

using Bradford assay. Then, 20 μg protein from each sample were reduced with 4 mM DTT (60°C for 30 min), modified with 12 mM iodoacetamide in 100 mM ammonium bicarbonate (in the dark, room temperature for 30 min) and digested by dilution with water to 2 M urea, 25 mM ammonium bicarbonate at a 1:50 enzyme-to-substrate ratio, overnight at 37°C. An additional second digestion with trypsin was done for 4 additional hours at 37°C.

Mass spectrometry analysis

The tryptic peptides were desalted using C18 tips (Harvard) dried and re-suspended in 0.1% formic acid. The peptides were resolved by reverse-phase chromatography on 0.075 x 180-mm fused silica capillaries (J&W) packed with C18 Reprosil-Aqua 3.5 μm reversed phase material (Dr Maisch GmbH, Germany). The peptides were eluted with linear 180 minutes gradient of 5 to 28%, 15 minutes gradient from 28 to 95%, and 10 minutes at 95% acetonitrile with 0.1% formic acid in water at flow rates of 0.15 μl/min. Mass spectrometry was performed by Q-ExactivePlus mass spectrometer (Thermo-Fisher Scientific, San Jose, CA) in a positive ion mode (m/z 350–1800, resolution 70,000) using repetitively full MS scan followed by collision induces dissociation (HCD) of the 10 most dominant ions (1 to 7 charges) selected from the first full MS scan. A dynamic exclusion list was enabled with exclusion duration of 20s.

The MS raw data from all the biological repeats were analyzed using the MaxQuant software[1] (version 1.5.2.8) for peak picking and quantitation, followed by identification using the Andromeda search engine (vs. the *Danio rerio* section- from Uniprot database), with a mass tolerance of 20 ppm for the precursor masses and fragment ions. Methionine oxidation carbamidomethyl on cysteine and propionamide on Cys, His, or Lys were set as variable post-translational modifications. Minimal peptide length was set to six amino acids, and a maximum of two miscleavages was allowed. To eliminate identifications from the reverse database and common contaminants, peptide- and protein-level false discovery rates (FDRs)

were filtered to 1% using the target–decoy strategy. MaxQuant software was used for quantitative analysis, based on extracted ion currents (XICs) of peptides, enabling quantitation from each LC-MS/MS run for each peptide identified in any of experiments. Only proteins that were identified with at least two peptides are listed. The LC-MS data were transformed to log2 intensities. Missing values were replaced with 18 (on the logarithmic scale), which correspond to the lowest intensity detected. *T*-test with permutation-based FDR (with 250 randomization, threshold value = 0.05) was done using Perseus 1.5.2.4 software[2]. GO and KEGG annotation enrichment was used to describe biological functions of the identified DEPs.

BBB-PAMPA

BBB-PAMPA Permeability Assay was performed by Creative Bioarray (Shirley, NY, USA) using a protocol based in[3], and propranolol was used as positive control. Briefly, the donor solutions of NAC (10 μM, 150 μL in PBS/DMSO 19:1) were added to each well of the donor plate, whose PVDF membrane was precoated with 5 μL of 1% brain polar extract (porcine)/dodecane mixture. Then, 300 μL of PBS were added to each well of the PTEF acceptor plate. The donor and acceptor plates were combined together and incubated for 4h at room temperature with shaking at 300 rpm. In each plate, NAC and the positive control were tested in duplicate. After incubation acceptor samples were prepared by mixing 270 μL of the solution from each acceptor well with 130 μL acetonitrile containing internal standard. Donor samples were prepared by mixing 20 μL of the solution from each donor well with 250 μL PBS and 130 μL acetonitrile containing internal standard. Then, NAC concentrations in the acceptor and the donor wells were analyzed by LC-MS/MS. The permeability rate (P_e in nm/s) was calculated with the following equation:

$$P_e = C\ x\ \left(-\ln\left(1-\frac{[drug]_{acceptor}}{[drug]_{equilibrium}}\right)\right) x\ 10^7$$

where $C = \left(\frac{VD\ x\ VA}{(VD+VA) x\ Area\ x\ time}\right)$,

$[drug]_{\text{equilibrium}}=([drug]_{\text{donor}}\ \text{x}\ VD + [drug]_{\text{acceptor}}\ \text{x}\ VA)/(VD+ VA)$

$[drug]_{\text{acceptor}} = (\text{Aa/Ai x DF})_{\text{acceptor}}$

$[drug]_{\text{donor}} = (\text{Aa/Ai x DF})_{\text{donor}}$

VD=0.15 mL; VA=0.30 mL; Area=0.28 cm^2; time=14400s.

Aa/Ai: Peak area ratio of NAC and internal standard; DF: Dilution factor (13.5).

Finally, the permeability of the tested compounds was classified by their P_e as high (P_e >10 nm/s), moderate (1 < P_e < 10) or low (P_e <1).

Analysis of NAC in brain samples

The extraction of NAC from zebrafish brain samples was adapted from previous published methods about the determination of metabolites in adults and larvae of zebrafish[4-6]. Five hundred microliters of a MeOH:H_2O (90:10) mixture was added to each zebrafish brain and shaken using a vortex mixer. Then, three stainless steel beads (3 mm diameter) were placed into each sample and were homogenized using a bead mill homogenizer (TissueLyser LT, Qiagen, Hilden, Germany) at 50 oscillations per min during 90 s. After this, samples were shaken for 20 min in a vibrating plate at 4°C and centrifuged for 20 min at 13,000 rpm, also at 4°C. The supernatant was filtered using 0.20 µM PTFE filters (DISMIC -13 JP, Advantec, Toyo Roshi Kaisha, Ltd., Japan) and kept in amber chromatographic vials at –80°C until LC-MS/MS analysis.

Moreover, the determination of NAC in the brains of the zebrafish samples was done using UPLC-MS/MS system (Waters, USA). To identify NAC, acquisition was performed in positive

electrospray ionization (ESI+) and acquired using selected reaction monitoring (SRM) mode with two transitions from precursor ion to product ions. Final transitions used as well as optimized cone voltage and collision energies are given in Supplementary Table ST1. Chromatographic conditions were the same as in the previous section ("*Analysis of NAC stability*").

Quality assurance of the method for determining NAC in brain extracts

Calibration standards were prepared in a 50:50 mixture of MeOH with 0.1% HCOOH and HPLC water with 0.1% HCOOH, as the samples. Calibration was performed over a concentration range from 0.005 to 1 ng/µL with seven calibration points using external calibration. Instrumental detection limit (IDL) of NAC was determined using the lowest concentration standard solution at 0.005 ng/µL that yielded a S/N ratio equal to 3, and method detection limit (MDL) was calculated in the same way, using zebrafish brain samples spiked at 500 ng. Solvent blanks did not contain any of the investigated analytes, indicating no carryover effect during the LC-MS/MS runs. Therefore, recovery studies were performed with five replicates, using zebrafish brain samples spiked at 500 ng of NAC, subtracting the possible traces of target compound with matrix blanks. Finally, the matrix effect (ME) was assessed by comparing the peak area of NAC from the spiked brain of zebrafish with the peak area of NAC from the standard solution at the same concentration in methanol (N = 5), following the equation:

$$ME(\%) = \frac{A - B}{C}\ x\ 100$$

where A is the peak area of NAC from spiked brain of zebrafish; B is the peak area of NAC from non-spiked brain of zebrafish; and C is the peak area of NAC in methanol.

Supplementary Table ST1 displays the quality parameters obtained by LC-MS/MS. External standard calibration was used obtaining good correlation coefficients (R^2) over 0.99 for NAC

in a range from 0.005 to 1 ng/µL. IDL was 2.05 pg and MDL 3.36 pg/mg brain. On the other hand, the recovery of NAC in the extraction procedure was excellent (101% ± 7%) and no matrix effect was obtained (91% ± 3%).

Novel tank test

The NTT, used to assess locomotor activity and anxiety, was performed using an experimental setup allowing monitoring and recording 2 fish simultaneously. The NTT was performed in two experimental tanks (20 cm length, 20 cm width, 25 cm height) containing 7 L (20 cm height) fish water at 28°C. LED backlight illumination (GP-G2, Quirumed, Spain) located behind the tank provided uniform illumination for video-recording. Control and ACR-exposed fish were tested in the standard 6-min NTT. Each trial was video-recorded (AVI format, 30 fps) with the uEye Cockpit software (version 4.90; IDS GmbH, Germany) controlling a GigE camera (UI-5240CP-NIR-GL, IDS GmbH, Germany) mounted in front of the experimental tank. In order to avoid any potential tank effect, experimental group assigned to each tank was switched between trials. After the recording was complete, the videos were analyzed by Ethovision XT 13.0.

Total glutathione determination

Brain tissue was homogenized in 5 % ice cold trichloroacetic acid (TCA), prepared in 0.1 mM Phosphate Buffer pH 7.4, at 50 mg/mL (weight:volume) proportion. The homogenates were then centrifuged at 4°C and 12,000 rpm for 5 min and the supernatant was collected for total glutathione (GSx) measurement[7].

Total glutathione content (GSx = GSH + 2GSSG) was determined in a Synergy 2 Multi-Mode Microplate Reader (BioTek® Instuments – Vermont, USA) microplate reader with a sensitive and specific assay using a β-nicotinamide adenine dinucleotide phosphate, reduced tetrasodium salt (NADPH) dependent recycling reaction of reduced glutathione (GSH) with

5,5-dithiobis(2-nitrobenzoic acid) (DTNB), forming a GS-TNB complex in the presence of excess glutathione reductase (GR)[7,8]. The final concentration of reagents was 0.21 mM NADPH, 0.6 mM DTNB and 0.175 U/mL GR in the presence of 0.05 mM triethanolamine prepared in phosphate buffer. Triethanolamine stabilizes pH to 6-7 to avoid autoxidation of reduced glutathione (GSSG). GSSG was used for GSx standard, where 1 μM of GSSG is equivalent to 2 μM of GS[7]. Standards were prepared and measured under the same conditions as samples. Samples and standards were incubated for 30 minutes at 25°C to allow formation of the GS-TNB complex and were then measured at 412 nm. Total GS in samples was extrapolated using the standard curve and final results were expressed as nmol GSx/g tissue ww (wet weight).

Acylase I activity

Acylase I activity was determined by measuring the formation of L-methionine by deacetylation N-acetyl-L-methionine (NAM) using fluorescamine as fluorescent probe[9]. Zebrafish brain, gill and gut tissue were homogenized in 50 mM Phosphate buffer pH 7.4 containing 1 mM of EDTA at a 18 and 20 mg/mL tissue buffer volume ratio, for brain and both gill and gut tissue, respectively. Homogenates were centrifuged at 12,000 g for 10min (4°C) and the supernatant was collected to enzyme activity measurement. The reaction mixture consisted of 75 μL of sample mixed with 175 μL of 11 mM NAM prepared in phosphate buffer, and incubated for 1 hour at 28°C acylase I-catalyzed deacetylation of N-acetyl-L-cysteine and S-alkyl-N-acetyl-L-cysteines C. After incubation, the reaction was stopped by adding 50 μL of 20% trichloroacetic acid (TCA). Sample blanks were prepared by adding TCA before NAM. The mixture was allowed to stand for 30 min on ice and then centrifuged for 5 min at 2,500 g (4°C). Then, 50 μL of the resulting supernatant was added into each well, of a black microplate, in triplicate, followed by 200 μL of fluorescamine

(0.303mg/ml, acetone) and the fluorescence intensity (390 nm excitation, 475 nm emission) was measured after 15min incubation at room temperature in a Synergy 2 Multi-Mode Microplate Reader(BioTek® Instuments – Vermont, USA). Sample concentration of L-methionine was extrapolated using a L-methionine standard curve (1-50 μM). Results were represented as pmol/min/mg of protein. Total protein concentrations were measured using the Bradford method with bovine serum albumin (BSA) as a standard[10].

GCL activity and GSx levels

The activity of glutamate cysteine ligase (GCL) activity was measured with a fluorescence based microplate assay, described by White et al.[11], based on the detection by reading the fluorescence generated by the complex that is formed by conjugation of naphthalene dicarboxyaldehyde (NDA) and γ-glutamylcysteine (γ-GC). Briefly, zebrafish brain and intestine were homogenized in ice cold TES/SB buffer (20 mM Tris, 250 mM sucrose, 1 mM EDTA, 20 mM sodium borate, 2 mM serine, pH 7.4), at a 16.5 and 18 mg/ml tissue wet weight/buffer volume proportion for brain and intestine, respectively. Homogenates were then centrifuged at 12,000 g for 10 min (4°C) and supernatants were collected for enzyme activity measurement and protein determination. Next, an aliquot (100 µL), referred to as, reaction sample (RS), was mixed with equal volume of reaction cocktail (400 mM Tris, 20 mM L-glutamic acid, 2 mM EDTA, 20 mM Sodium Borate, pH 7.4) containing 40 mM ATP, freshly added. The reaction was initiated by adding 100 µL of 5 mM of L-cysteine, and incubated at 28°C for 15 min. During incubation period, GCL present in the sample catalyzes an ATP-dependent reaction that condensates cysteine and glutamate forming γ-GC. A second aliquot was used for base line measurement of GSx and referred to as sample blank (SB). The SB aliquot was submitted to the same incubation conditions as the RS, with the exception of blocking GCL activity by adding 100 µL of 200 mM sulfoalicylic acid (SSA) before adding L-

cysteine. After the incubation period, GCL activity in the RS was stopped by adding 100 µL of ice cold 200 mM SSA, and all aliquots were incubated on ice for 30min to allow protein precipitation. Samples were then centrifuged for 5 min at 2,500 g, 4°C. In a black microplate, 20µL of the resulting supernatant was placed in each well in triplicates (3 RS and 3 SB). Samples were diluted 2.25x with 50mM Tris-HCL Buffer pH 10. Next, 155 µL of NDA alkaline solution (50 mM Tris Buffer pH 10; 0.5 N NaOH and 10 mM 2,3-NDA in DMSO; v/v/v, 7/1/1), was added to each well. The plate was protected from light and allowed to incubate at room temperature for 30 min. The cyclic compounds, γ-GC-NDA or GS-NDA formed between NDA and γ-GC or GSH, respectively, was measured in a Synergy 2 Multi-Mode Microplate Reader (BioTek® Instuments – Vermont, USA) with wavelengths of 485 and 535 nm for excitation and emission, respectively. Since White et al[11] determined that fluorescence intensities of equimolar concentrations of γ-GC or GSH conjugated with NDA were essentially the same, a standard of GSH was used to extrapolate γ-GC equivalent concentration in samples. Results of γ-GCL activity are expressed as nmol of equivalent γ-GC formed/min/mg of protein, while GSx levels are represented as nmol GSx/g tissue wet weight (ww). Total protein concentrations were measured using the Bradford method with bovine serum albumin (BSA) as a standard[10].

Supplementary Tables

Supplementary Table ST1. LC-MS/MS optimized parameters for N-Acetylcysteine (NAC) and the corresponded quality parameters. C.V.: cone voltage (V); C.E.: collision energy.

Mass spectral characterization							
Molecular formula	Molecular weight	Precursor ion	C.V. (V)	Fragment ion 1	C.E. 1 (eV)	Fragment ion 2	C.E. 2 (eV)
$C_5H_9NO_3S$	163.19	164 $[M+H]^+$	30	122	7	76	19
**Quality parameters**							
Linearity (ng μL^{-1})	Calibration type	F	R^2	IDL (pg)	%Recovery ±RSD (N=5)	%M.E. ±RSD (N=5)	MDL (pg/mg brain)
0.005-1	External	1e6	0.9998	2.05	101±7	91±3	3.36

Supplementary Table ST2. NAC content in the NAC and NAC+ACR working solutions. For each NAC concentration and time, differences in the NAC content between the NAC and NAC+ACR working solution were tested by using the Mann-Whitney test, and the probability (p) is indicated in the table. Three replicates per condition.

	**0.3 mM NAC** (mean ±SE, mM)	**0.3 mM NAC+ACR** (mean ±SE, mM)	***p***
**Time 0**	0.30 ± 0.02	0.30 ± 0.03	1.000
**Time 24h**	0.16 ± 0.07	0.13 ± 0.01	1.000

	**0.75 mM NAC** (mean ±SE, mM)	**0.75 mM NAC+ACR** (mean ±SE, mM)	***p***
**Time 0**	0.75 ± 0.02	0.75 ± 0.01	1.000
**Time 24h**	0.57 ± 0.06	0.56 ± 0.07	1.000

Supplementary Table ST3. One way ANOVA assessing effects of each experimental group over the variance of zebrafish behavioral endpoints.

	df1	df2	F	Sig.
**Total Distance**	3	82	15.198	6.76×10^{-8}
**Distance in the top**	3	82	23.451	5.92×10^{-11}
**Distance in the bottom**	3	82	7.371	0.00020
**Time in the top**	3	82	33.706	3.94 x 10-14
**Time in the bottom**	3	82	20.112	8.82×10^{-10}
**Latency to the top**	3	77	23.630	7.85×10^{-11}
**Freezing time**	3	85	13.117	4.46×10^{-7}
**Freezing bouts**	3	85	6.798	0.00038

df1: df between groups; df2: df total

Supplementary Figures

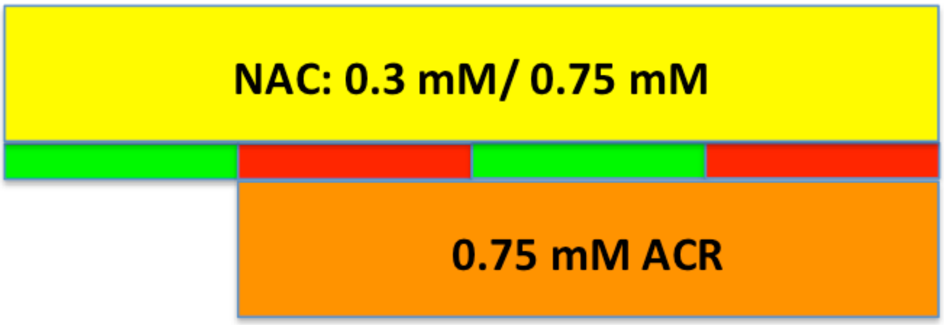

Supplementary Figure S1. Experimental design of NAC and ACR co-exposure experiments. For analyzing the protective effect of NAC, adult zebrafish was pre-treated with 0.3 or 0.75 mM NAC for 24 h and then co-exposed to a mixture of 0.75 mM ACR plus NAC at the same concentration used for the pre-treatment, for an additional 72h.

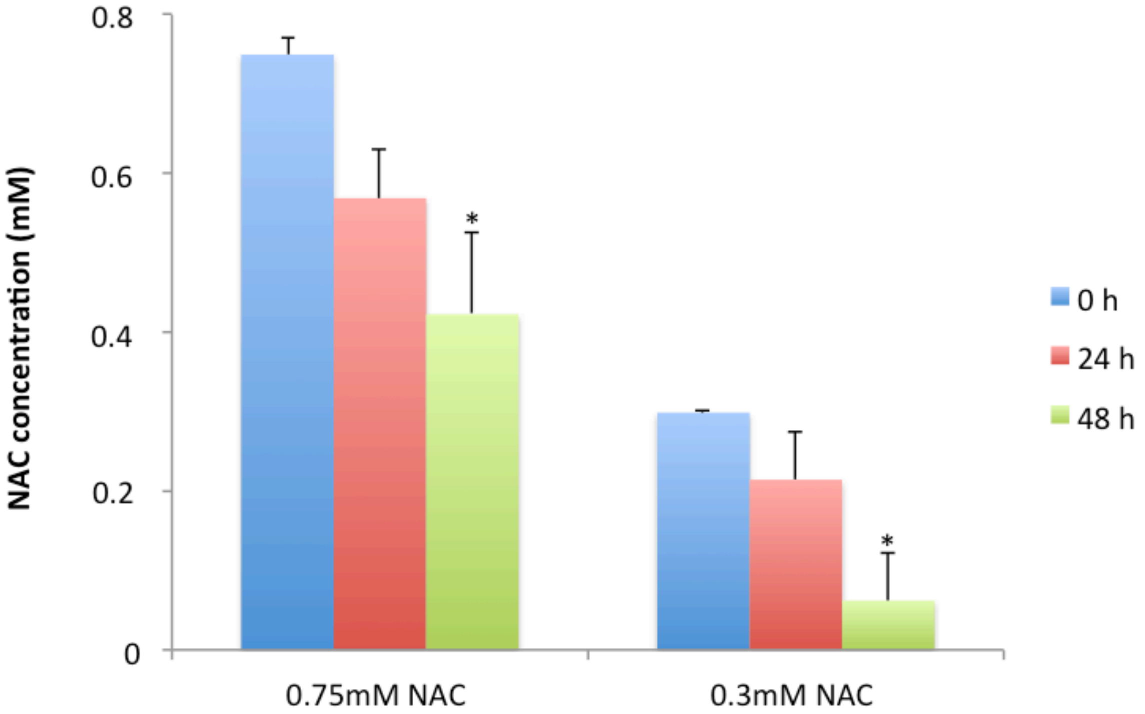

Supplementary Figure S2. Time-course of NAC degradation. There is a time-dependent decrease in the NAC content in the 0.74 mM and 0.3 mM NAC working solutions.

* $p<0.05$, Kruskal Wallis test, n=3

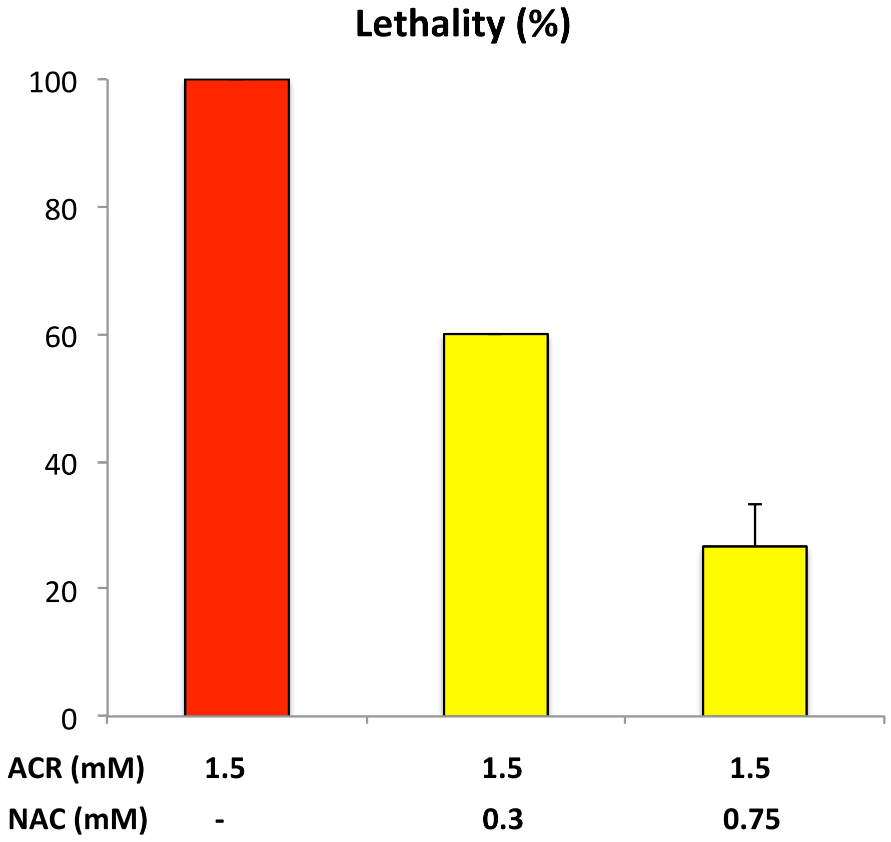

Supplementary Figure S3. Lethality rates of zebrafish treated with 1.5 mM ACR for 72 h, or pretreated with NAC (0.3 and 0.75 mM) for 24h and then co-exposed to NAC and 1.5 mM ACR for 72h. Data reported as mean ± SEM, from 3 independent experiments (n=15)

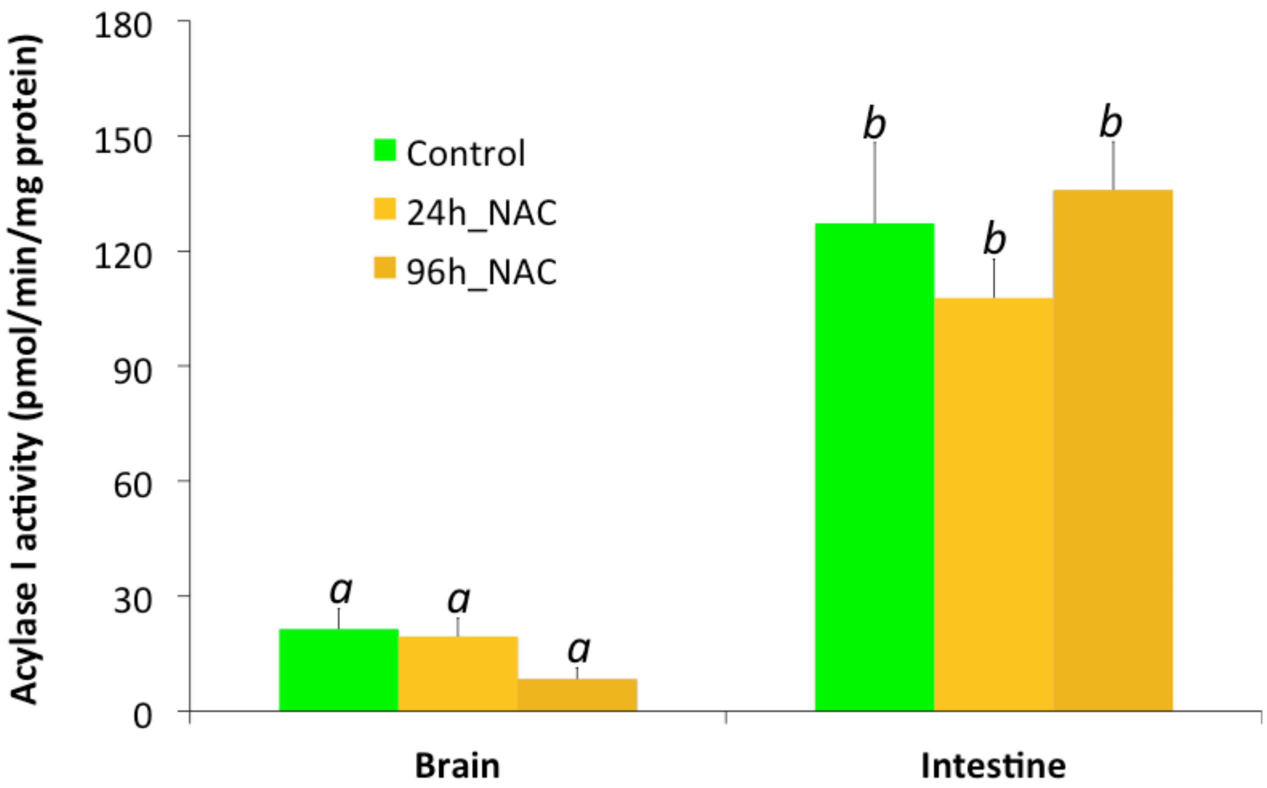

Supplementary Figure S4. Acylase I activity in brain and intestine: effect of NAC. Basal acylase I activity in the intestine is significantly higher than in the brain (p<0.001). However, NAC has not a significant effect of acylase activity in any of these organs (p>0.05). Data reported as mean ± SEM. Different letters indicate significant (*P*<0.05) differences following one-way ANOVA and Tukey's multiple-comparison test. Data from 3 independent experiments (n=9).

Supplementary Discussion

ACR-treatment resulted in the covalent modification of 146 proteins in cysteine, lysine or histidine residues. Results on the modified proteins obtained in this study are very consistent with previous reports on zebrafish[4], and rodent[12] brains. Thus, modified proteins include important proteins involved in the recycling of the synaptic vesicles (cplx2, syn1, syn2b, sanp25a, syt7b, zgz:92912 and rtn1a), neurofilaments (nefma, nefmb), microtubule associated proteins (MAPs; map1aa, map1ab, map2, map6b, gphna), and some proteins essential in redox control (txn, zgc:56493, prdx5, msrb2). Many relevant proteins of the GABAergic system were also found modified, including gad1b and gad2, involved in the synthesis of GABA[13], slc32a1, a vesicular GABA transporter[14], phf24, a regulator of GABAergic system[15], or slc6a11b, also known as GABA transporter 3[16]. Interestingly, some of the proteins protected by NAC are directly involved in the synaptic function (cplx2, zgc:92912, rtn1a, gphna, got1, gad1b)[14,17,18], or redox regulation (txn1)[19].

Supplementary References

1 Cox, J. & Mann, M. MaxQuant enables high peptide identification rates, individualized ppb-range mass accuracies and proteome-wide protein quantification. *Nature Biotechnology* **26**, 1367-1372 (2008).

2 Tyanova, S. *et al.* The Perseus computational platform for comprehensive analysis of (prote) omics data. *Nature Methods* **13**, 731-740 (2016).

3 Rabal, O. *et al.* Detailed exploration around 4-aminoquinolines chemical space to navigate the lysine methyltransferase G9a and DNA methyltransferase biological spaces. *Journal of Medicinal Chemistry* **61**, 6546-6573 (2018).

4 Faria, M. *et al.* Acrylamide acute neurotoxicity in adult zebrafish. *Scientific Reports* **8**, 7918 (2018).

5 Gómez-Canela, C., Prats, E., Tauler, R. & Raldúa, D. Analysis of neurobehavioural data by chemometric methods in ecotoxicological studies. *Ecotoxicology and Environmental Safety* **145**, 583-590 (2017).

6 Gómez-Canela, C. *et al.* Comprehensive characterization of neurochemicals in three zebrafish chemical models of human acute organophosphorus poisoning using liquid chromatography-tandem mass spectrometry. *Analytical and Bioanalytical Chemistry* **410**, 1735-1748 (2018).

7 Baker, M. A., Cerniglia, G. J. & Zaman, A. Microtiter plate assay for the measurement of glutathione and glutathione disulfide in large numbers of biological samples. *Analytical Biochemistry* **190**, 360-365 (1990).

8 Pena-Llopis, S., Pena, J., Sancho, E., Fernandez-Vega, C. & Ferrando, M. Glutathione-dependent resistance of the European eel Anguilla anguilla to the herbicide molinate. *Chemosphere* **45**, 671-681 (2001).

9 Uttamsingh, V., Keller, D. & Anders, M. Acylase I-catalyzed deacetylation of N-acetyl-L-cysteine and S-alkyl-N-acetyl-L-cysteines. *Chemical Research in Toxicology* **11**, 800-809 (1998).

10 Bradford, M. M. A rapid and sensitive method for the quantitation of microgram quantities of protein utilizing the principle of protein-dye binding. *Analytical Biochemistry* **72**, 248-254 (1976).

11 White, C. C., Viernes, H., Krejsa, C. M., Botta, D. & Kavanagh, T. J. Fluorescence-based microtiter plate assay for glutamate–cysteine ligase activity. *Analytical Biochemistry* **318**, 175-180 (2003).

12 Barber, D. S., Stevens, S. & LoPachin, R. M. Proteomic analysis of rat striatal synaptosomes during acrylamide intoxication at a low dose rate. *Toxicological Sciences* **100**, 156-167 (2007).

13 Bosma, P. T. *et al.* Multiplicity of glutamic acid decarboxylases (GAD) in vertebrates: molecular phylogeny and evidence for a new GAD paralog. *Molecular Biology and Evolution* **16**, 397-404 (1999).

14 Horzmann, K. & Freeman, J. Zebrafish Get connected: investigating neurotransmission targets and alterations in chemical toxicity. *Toxics* **4**, 19 (2016).

15 Kuramoto, T. *et al.* Identification of Candidate Genes for Generalized Tonic–Clonic Seizures in Noda Epileptic Rat. *Behavior Genetics* **47**, 609-619 (2017).

16 Scimemi, A. Structure, function, and plasticity of GABA transporters. *Frontiers in Cellular Neuroscience* **8**, 161 (2014).

17 Takamori, S. *et al.* Molecular anatomy of a trafficking organelle. *Cell* **127**, 831-846 (2006).

18 Tretter, V. E. *et al.* Gephyrin, the enigmatic organizer at GABAergic synapses. *Frontiers in Cellular Neuroscience* **6**, 23 (2012).

19 Silva-Adaya, D., Gonsebatt, M. E. & Guevara, J. Thioredoxin system regulation in the central nervous system: experimental models and clinical evidence. *Oxidative Medicine and Cellular Longevity* **2014**, 590808 (2014).
